# Efficient Synthesis of Key Chiral Intermediate in Painkillers (R)-1-[3,5-Bis(trifluoromethyl)phenyl]ethanamine by Bienzyme Cascade System with R-ω-Transaminase and Alcohol Dehydrogenase Functions

**DOI:** 10.3390/molecules27217331

**Published:** 2022-10-28

**Authors:** Yuan Lu, Jinmei Wang, Haobo Xu, Chuyue Zhang, Pengpeng Cheng, Lihua Du, Lan Tang, Jinghua Li, Zhimin Ou

**Affiliations:** 1 College of Pharmaceutical Science, Zhejiang University of Technology, Hangzhou 310014, China; 2 College of Biotechnology and Bioengineering, Zhejiang University of Technology, Hangzhou 310014, China

**Keywords:** alcohol dehydrogenase, bienzyme cascade system, chiral amines, R-ω-transaminase, recombinant engineered bacteria

## Abstract

(R)-1-[3,5-bis(trifluoromethyl)phenyl]ethanamine, a key chiral intermediate of selective tetrodotoxin-sensitive blockers, was efficiently synthesized by a bienzyme cascade system formed by with R-ω-transaminase (ATA117) and an alcohol dehydrogenase (ADH) co-expression system. Herein, we report that the use of ATA117 as the biocatalyst for the amination of 3,5-bistrifluoromethylacetophenone led to the highest efficiency in product performance (enantiomeric excess > 99.9%). Moreover, to further improve the product yield, ADH was introduced into the reaction system to promote an equilibrium shift. Additionally, bienzyme cascade system was constructed by five different expression systems, including two tandem expression recombinant plasmids (pETDuet-ATA117-ADH and pACYCDuet-ATA117-ADH) and three co-expressed dual-plasmids (pETDuet-ATA117/pET28a-ADH, pACYCDuet-ATA117/pET28a-ADH, and pACYCDuet-ATA117/pETDuet-ADH), utilizing recombinant engineered bacteria. Subsequent studies revealed that as compared with ATA117 single enzyme, the substrate handling capacity of BL21(DE3)/pETDuet-ATA117-ADH (0.25 g wet weight) developed for bienzyme cascade system was increased by 1.50 folds under the condition of 40 °C, 180 rpm, 0.1 M pH9 Tris-HCl for 24 h. To the best of our knowledge, ours is the first report demonstrating the production of (R)-1-[3,5-bis(trifluoromethyl)phenyl]ethanamine using a bienzyme cascade system, thus providing valuable insights into the biosynthesis of chiral amines.

## 1. Introduction

Chiral amines are indispensable building blocks in active pharmaceutical ingredients, agrochemicals, and bioactive natural products [1,2]. Biocatalytic asymmetric amination has emerged as an attractive alternative to traditional chemical methods for the synthesis of chiral amines [3]. Transaminases (TAs) have been used as biocatalysts for the synthesis of optically pure amines owing to their excellent stereoselectivity, high yield, broad substrate specificity, environmental friendliness, and lack of redox cofactor recycling [4,5,6]. TAs catalyze the transfer of an amino group from an amino donor to a carbonyl moiety via a ping-pong bi-bi mechanism, utilizing pyridoxal-5′-phosphate (PLP) as a cofactor [7,8,9]. Regarding the synthesis of chiral amines, TAs are one of the most promising enzymes that provide a more concise route for reductive amination than their chemical counterparts [10]. Furthermore, they can be divided into α-TAs and ω-TAs, according to the position of the amino group that is transferred [11,12,13]. α-TAs transfer the amino group at the α-position of an amino acid, whereas ω-TAs act on the amino group at any position other than the α-position. In contrast to α-TAs, ω-TAs do not necessarily require a carboxylate group in their substrates, which makes them much more versatile for biocatalytic transformations than the α-TAs [14,15]. Recently, considerable efforts have been made to discover both (S)- and (R)-selective ω-TAs by screening natural sources and protein engineering techniques [16,17,18]. Before the first report of (R)-selective ω-TAs (R-ω-TAs) by Iwasaki et al. [19], the enzymatic synthesis of (R)-amines was attempted using the kinetic resolution of racemic amines employing (S)-selective ω-TAs (S-ω-TAs) [20]. However, asymmetric synthesis of (R)-amines using R-ω-TAs has been reported in multiple studies as their theoretical yield can be 100% [21,22,23]. A famous example in the biopharmaceutical industry is the production of sitagliptin (an oral hypoglycemic agent) using engineered R-ω-TA [24,25]. In the process of developing this biocatalyst, the substrate-binding pocket of R-ω-transaminase (ATA117, *Arthrobacter* sp. KNK168) was incrementally adapted to prositagliptin ketone from wild-type ATA117 (ATA117 WT), yielding a highly active R-ω-TA variant (ATA117-Rd11) [26,27]. Although ATA117-Rd11 has been successfully employed in the production of sitagliptin, the substrate scope of the enzyme still requires further improvement to increase its applicability. Consequently, a series of structural analogs were selected to explore the potential substrates of ATA117-Rd11, according to the skeletal structure of the sitagliptin precursor ketone [12,13].

**Figure 1:**
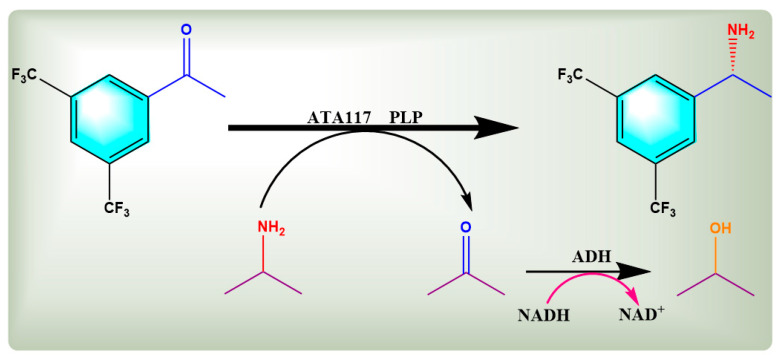
Schematic diagram of the reaction mechanism of ATA117 and ADH double enzymatic cascade reaction for amination of BPO to R-BPA.

In practical applications, owing to the unfavorable thermodynamic equilibrium, the suprastoichiometric addition of isopropylamine and in situ removal of the corresponding acetone by-product are typically required to shift the reaction equilibrium in the desired direction [28,29]. Accordingly, various process control strategies have been developed to remove the products in situ, among which cascade reactions employing additional biochemical reactions of the side products have garnered much attention [30,31,32]. Multienzyme cascade systems that catalyze biological cascade reactions are the result of natural evolution. Recent studies suggest that the specific microenvironment created by enzyme co-localization in confined and/or crowded spaces is a major determinant for increased reaction efficiency, rather than often-cited substrate channeling induced by spatial proximity of enzymes [33,34]. In those microenvironments, the intermediates are likely ‘pseudo’-trapped through reversible interactions (i.e., ionic, Van der Waals, hydrogen bonds) that hamper their dilution into the reaction bulk [35]. In this study, a bienzyme cascade system of ATA117 and alcohol dehydrogenase (ADH, *Saccharomyces cerevisiae* S288C) was constructed through co-expression strategies. Equilibrium displacement was enabled by removing the coproduct acetone employing ADH in combination with NADH [36,37]. The ADH features a very narrow substrate spectrum and is therefore unable to accommodate medium or large groups (>C3) [38]. The ATA117/ADH co-expression system was used for the first time to catalyze the asymmetric amination of 3,5-bistrifluoromethylacetophenone (BPO) and prepare (R)-1-[3,5-bis(trifluoromethyl)phenyl]ethanamine (R-BPA) [39]. R-BPA is a chiral intermediate of selective tetrodotoxin-sensitive blockers, which have various therapeutic uses in pain management. The ADH was introduced in this system to convert the acetone by-product and improve the ammonia conversion efficiency (Figure 1).

The co-expression of multiple enzymes in one host due to the sharing of the protein synthesis machinery could decrease enzyme production costs compared to individual enzyme expression in multiple hosts followed by enzyme cocktailing. Therefore, more attention has been devoted to optimizing the co-expression method of the two enzymes used to construct a bienzyme cascade system, aiming to improve its catalytic efficiency and yield in transamination reactions in our present study. This was achieved by adjusting the co-expression mode of the two genes to obtain a high-efficiency bienzyme cascade system. The co-expression system was constructed using a dual-gene tandem expression system (pACYCDuet-ATA117-ADH and pETDuet-ATA117-ADH) and a dual-plasmid expression system (pETDuet-ATA117/pET28a-ADH, pACYCDuet-ATA117/pET28a-ADH, and pACYCDuet-ATA117/pETDuet-ADH). After the successful construction of the above five dual-gene co-expression systems (Table 1), the optimal system was selected from the five recombinant engineered bacteria of the co-expression systems and the bienzyme coupling system consisting of ATA117 and an ADH-free enzyme from a single expression system due to the higher specific enzyme activity of ATA117 (0.18 U/mg). At 40 °C, 207.8 mM substrate BPO was fully converted to the product R-BPA with high selectivity (99.9%) using BL21(DE3)/pETDuet-ATA117-ADH as a catalyst. In metabolic engineering and synthetic biology, multiple heterologous genes are usually introduced into one host to implement a synthesis of a desired compound while the precise control of their expression levels is of importance to avoid low expression of a rate-limiting enzyme or over-expression of non-rate-limiting enzymes [40]. The study of the coordinated co-expression levels of ATA117 and ADH genes in the same host cell provides a reference for the preparation of drug intermediate BPA.

## 2. Results and Discussion

### 2.1. Expression and Purification of ATA117 and ADH from Single Expression System

**Figure 2:**
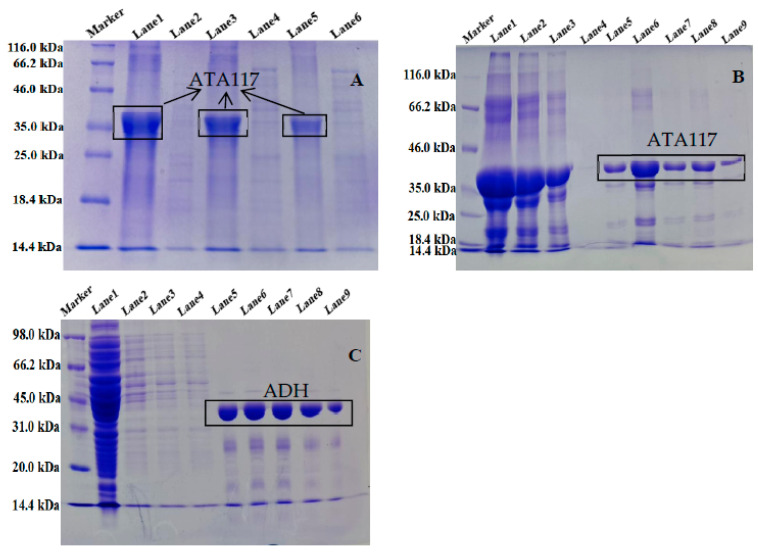
The SDS-PAGE analysis of ATA117 expressed by bacteria transformed with pETDuet-ATA117, pET28a-ATA117, pACYCDuet-ATA117 (**A**), the purification of ATA117 expressed by bacteria transformed with pETDuet-ATA117 (**B**), and the purification of ADH expressed by bacteria transformed with pET28a-ADH (**C**). ((**A**): Lane 1: Supernatant of pETDuet-ATA117; Lane 2: Precipitation of pETDuet-ATA117; Lane 3: Supernatant of pET28a-ATA117; Lane 4: Precipitation of pET28a-ATA117; Lane 5: Supernatant of pACYCDuet-ATA117; Lane 6: Precipitation of pACYCDuet-ATA117. (**B**): Lane 1: ATA117 stock solution; Lane 2: Flow-through solution; Lane 3: ATA117 initial wash solution; Lane 4: ATA117 final wash solution; Lane 5-Lane 9: ATA117 eluent 1–5. (**C**): Lane 1: ADH stock solution; Lane 2-Lane 4: ADH wash solution; Lane 5-Lane 9: ADH eluent 1–5).

To study the expression of ATA117 in *E. coli* BL21(DE3), three recombinant plasmids (pETDuet-ATA117, pACYCDuet-ATA117, and pET28a-ATA117) containing the coding sequences for *ATA117* were constructed. After inducing the expression under their respective optimal conditions (Table 2), the enzyme was purified to more than 99% electrophoretic homogeneity using Ni-NTA agarose affinity chromatography. Furthermore, the SDS-PAGE analysis (Figure 2A) showed a single band with a molecular mass of ~38 kDa, which is in agreement with the predicted molecular weight of ATA117 based on its amino acid sequence. The theoretical size of the ATA117 target protein was predicted to be 36.5 kDa, while that of the fusion protein was 38.5 kDa. Additionally, the purified ATA117 exhibited a specific activity of 0.18 U/mg toward BPO. Accordingly, the protein expression of the three recombinant plasmids containing the ATA117 single gene was shown in Figure 2, and their activity comparison is shown in Table 2. pACYCDuet-1, pET28a, and pETDuet-1 are all expression plasmids. Among them, pACYCDuet-1 is a low-copy plasmid, whereas pET28a and pETDuet-1 are high-expression plasmids. Our results showed that the three recombinant plasmids pACYCDuet-ATA117, pET28a-ATA117, and pETDuet-ATA117 successfully expressed the target protein (Figure 2A). However, among the three recombinant plasmids, pETDuet-ATA117 showed the highest level of soluble protein expression, whereas a small portion of the target protein produced by BL21(DE3)/pET28a-ATA117 engineered bacteria was found to be insoluble. Although the protein expressed by the pET28a-ATA117 engineered bacteria was soluble, its expression level was significantly lower than that expressed by the pETDuet-ATA117 engineered bacteria. Among the three, the expression level of ATA117 by the pACYCDuet-ATA117 engineered bacteria was the lowest, which might be attributed to its low copy numbers. Subsequently, by measuring the activity of ATA117, it was found that ATA117 was best expressed in bacteria transformed with pETDuet-ATA117, with its activity being determined to be 1.28 times and 2 times that of bacteria transformed with pET28a-ATA117 and pACYCDuet-ATA117, respectively. Consequently, the pETDuet-1 plasmid was determined to be the most suitable for the efficient expression of ATA117. The purification of ATA117 expressed by *E. coli* BL21(DE3) transformed with pETDuet-ATA117 was shown in Figure 2B. The results demonstrated that the His-tag was successfully expressed by the engineered bacteria, and a single band with the same theoretical size as predicted for ATA117 was obtained by Ni-NTA column purification. Similarly, using *E. coli* BL21(DE3), two recombinant plasmids (pETDuet-ADH and pET28a-ADH) were successfully constructed to induce the expression of ADH under their respective optimal conditions. Post ultrasonication, the product was purified using a Ni-NTA agarose affinity chromatography, and its expression and purification were detected using SDS-PAGE. The results demonstrated that the His-tag was successfully expressed by the engineered bacteria and a single band with the same theoretical size as predicted for ADH was obtained by Ni-NTA column purification (Figure 2C). Subsequent assessment of the enzymatic activities of ADH expressed by bacteria transformed with pETDuet-ADH and pET28a-ADH revealed that there was a minute difference between their activities, with the activity of ADH from pETDuet-ADH transformed bacteria being slightly higher (Table 2).

### 2.2. Catalytic Properties of Purified ATA117 Expressed by the E. coli BL21(DE3)/pETDuet-ATA117 Recombinant Engineering Bacteria

To expand the substrate spectrum of ATA117, the reactivities of 13 different substrates (amino acceptors) were compared. The results revealed that although the ATA117 catalyzed reactions using methyl acetoacetate, ethyl acetoacetate, and 3,5-bistrifluoromethylacetophenone as substrates led to good yields; the corresponding enantiomeric excess was slightly different (Table 3). The substrate spectrum of ATA117 is relatively wide. ATA117 can catalyze the transamination reaction of acetophenone, 3,5-bistrifluoromethylacetophenone, benzylacetone, and p-methoxyphenylacetone to phenylethylamine, 1-[3,5-bis(trifluoromethyl)phenyl]ethanamine, 4-phenyl-2-butylamine, and 1-(4-methoxyphenyl)propan-1-amine, respectively. Moreover, among them, the reaction with 3,5-bistrifluoromethylacetophenone as the substrate exhibited the dual advantage of substantial yield and better enantioselectivity. Consequently, it was further selected for subsequent studies.

**Figure 3:**
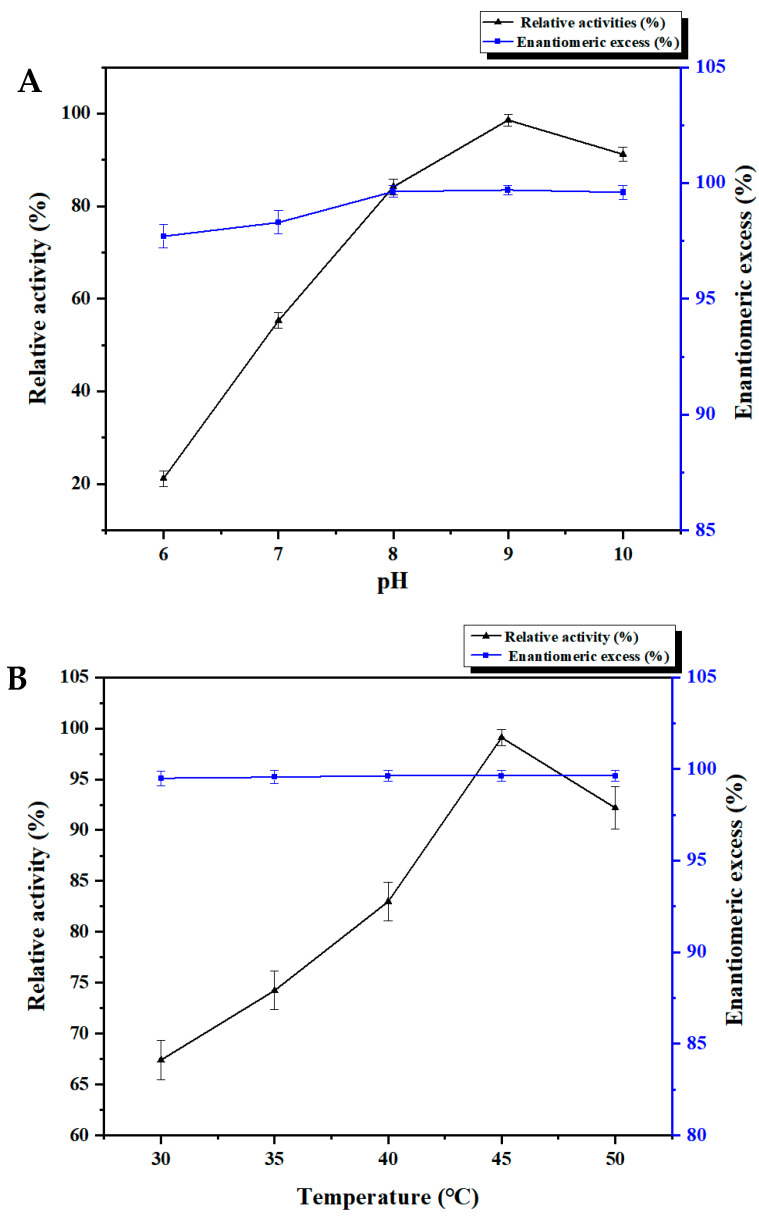

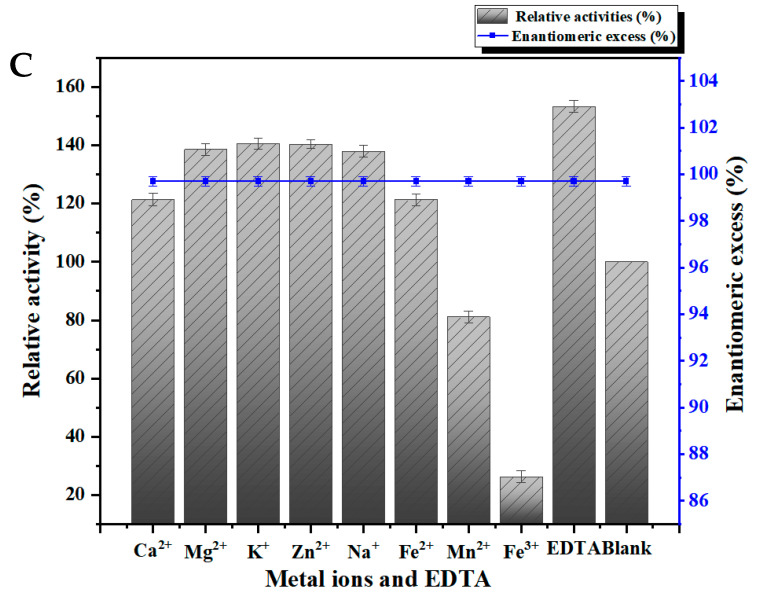
Effects of pH (**A**), temperature (**B**), metal ions and reagents (**C**) on the enzyme activity of the purified ATA117 expressed by the *E. coli* BL21(DE3)/pETDuet-ATA117 recombinant engineering bacteria. (A total of 2 mL of reaction system: substrate 166.6 mM, IPA 832.8 mM, PLP 1 mM, 15% DMSO, 2.7 U of transaminase pure enzyme aqueous solution, buffer to make up 2 mL volume.)

Additionally, we investigated the effect of pH in the range of pH 6.0–10.0 on the catalytic performance of ATA117. The results revealed that in the pH range of 6.0–9.0, the substrate conversion of ATA117 was found to be gradually increasing with pH. However, the same slightly decreased at pH 10.0 but remained high. Thus, our findings suggest that the pH of the reaction medium can affect the enzyme activity, which may be due to the change in the three-dimensional conformation of the enzyme active site due to a change in the pH of the reaction medium (Figure 3A). Moreover, the assessment of the relative activity trend indicated that the ATA117 is alkalophilic. The results showed that when the pH of the buffer solution was greater than 8.0, the relative activity was found to be controlled at >80% while all the products at this time were determined to be the target product R-BPA. As a result, 0.1 M Tris-HCl (pH 9.0) was determined to be the best buffer for this reaction system. The catalytic activity of an enzyme is significantly affected by temperature as it is lost or inhibited at high temperatures owing to protein denaturation. Accordingly, our results revealed that the relative activity of ATA117 was initially increased and subsequently decreased within a certain temperature range (30–50 °C) and reached a maximum at 45 °C (Figure 3B). 

Additionally, the effects of high concentrations of metal ions and EDTA on ATA117 activity were investigated (Figure 3C). The results demonstrated that, after the addition of different kinds of metal ions and EDTA, the relative activity of most of the reaction groups was improved to a certain extent (1.2–1.6 times), while the effect of 10 mM metal ion chelator EDTA was found to be the most effective (1.5 times). Moreover, the addition of 10 mM Mn^2+^ and Fe^3+^ inhibited the TA reaction efficiency to varying degrees, with Fe^3+^ exhibiting the greatest inhibitory effect. 

### 2.3. Comparison of ATA117 Activity from E. coli BL21(DE3)/pETDuet-ATA117 Whole Cells and Crude Enzyme Solution

According to the method described in Section 3.4.1, the activity of the whole cells obtained from the fermentation broth and the enzymatic broth after cell disruption was investigated. The results revealed that the whole cell-associated enzyme activity was 91% as compared with that of the crude enzyme solution obtained after disruption of the same amount of the cellular biomass. This finding is probably attributed to the intracellular location of ATA117, resulting in a beneficial effect on the ATA117 release post appropriate cell disruption method. This in turn may lead to more contact between the catalyst and the substrate, which is conducive to enzymatic activity. Accordingly, considering the maximum enzymatic activity, the usage of a sonication cytometer was considered the best method to disrupt the cells for obtaining the crude enzyme solution to complete the double-enzyme cascade reaction.

### 2.4. Asymmetric Synthesis of R-BPA from BPO by Free Bienzyme and Co-Expression Plasmid Engineered Bacteria

#### 2.4.1. Preparation of R-BPA by Bienzyme Cascade Reaction with Free Enzyme Mixture Containing ATA117 and ADH

**Figure 4:**
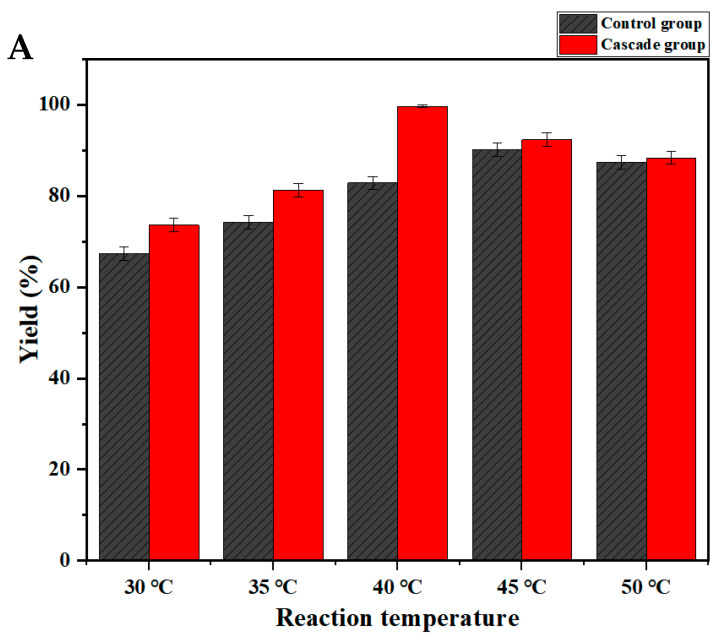

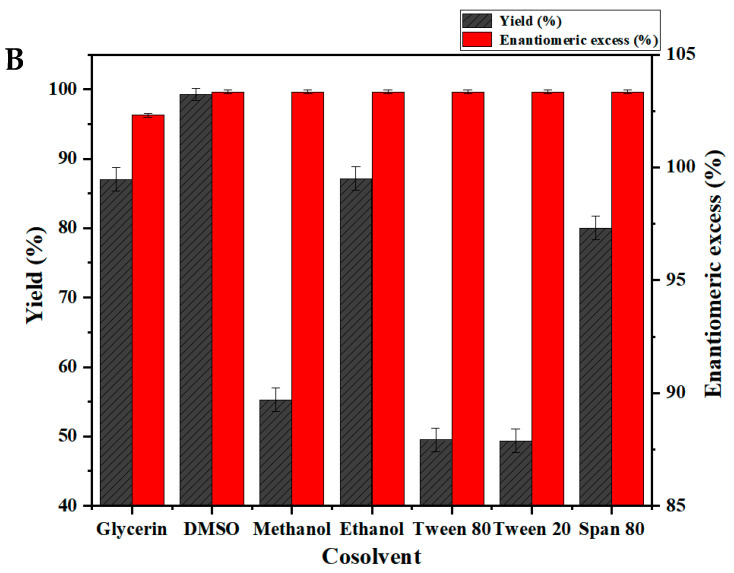
Effects of reaction temperature (**A**) and cosolvents (**B**) on the cascade reaction with free ATA117 and ADH expressed by single expression system.

While studying the production of R-BPA using a free bienzyme system, the addition of ADH, in the range of 30–40 °C, resulted in the evolution of the ATA117-mediated enzymatic reaction in the forward direction. The investigation showed that the IPA added in a superstoichiometric amount was converted to form a corresponding acetone by-product, while ADH catalyzes the reaction of acetone to isopropanol, which can facilitate the removal of the corresponding acetone by-product. The reaction was thus balanced to proceed in a forward direction thereby promoting the catalytic efficiency of the free bienzyme cascade reaction using free ATA117 and ADH. When the temperature was further increased to 50 °C, the catalytic advantage of the cascade reaction was no longer evident; the yield was slightly improved as compared with the control group while the ADH was hardly affected (Figure 4A). When the reaction temperature was 40 °C, the yield of the cascade group was 99.9%, while that of the control group was 82.9%. Consequently, a temperature of 40 °C was selected for the bienzyme-catalyzed cascade reaction system. Furthermore, the usage of different cosolvents exhibited noticeable differences in the solubility of the substrate. The results revealed that while the type of cosolvent mainly affected the yield to a certain extent, there was little effect on the enantiomeric excess of R-BPA (Figure 4B), which was controlled at a high level (≥96%). The data from the enzyme assays using Tween 20 and Tween 80 as cosolvents showed that there is little difference in the effect of Tween-based solvents on BPO solubilization. Additionally, an assessment of both the yield and enantiomeric excess (%) revealed that DMSO, ethanol, and glycerol are all suitable cosolvents for the asymmetric amination of BPO to R-BPA, with DMSO showing the best effect. Consequently, DMSO was chosen as the optimal cosolvent for the cascade reaction. The co-expression of multiple enzymes in one host due to the sharing of the protein synthesis machinery could decrease enzyme production costs compared to individual enzyme expression in multiple hosts followed by enzyme cocktailing. Therefore, the co-expression of the two enzymes in the same host was followed up.

#### 2.4.2. Preparation of R-BPA by Bienzyme Cascade System via Co-Expression Engineering Bacteria

**Figure 5:**
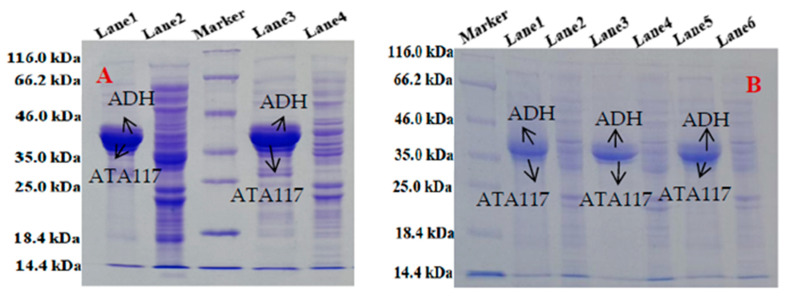
The protein expression of ATA117/ADH by the two tandemly expressing recombinant engineered bacteria (**A**) and three double-plasmid engineered bacteria (**B**). (**A**): Lane 1: Supernatant of pACYCDuet-ATA117-ADH, Lane 2: Precipitation of pACYCDuet-ATA117-ADH, Lane 3: Supernatant of pETDuet-ATA117-ADH, Lane 4: Precipitation of pETDuet-ATA117-ADH, (**B**): Lane 1: Supernatant of pACYCDuet-ATA117/pETDuet-ADH, Lane 2: Precipitation of pACYCDuet-ATA117/pETDuet-ADH, Lane 3: Supernatant of pACYCDuet-ATA117/pET28a-ADH, Lane 4: Precipitation of pACYCDuet-ATA117/pET28a-ADH, Lane 5: Supernatant of pETDuet-ATA117/pET28a-ADH, Lane 6: Precipitation of pETDuet-ATA117/pET28a-ADH).

Both pACYCDuet-1 and pETDuet-1 plasmids are *E. coli* protein dual-expression vectors with two multiple cloning sites. Therefore, they can be used to simultaneously express two target genes. Accordingly, *ATA117* and *ADH* were simultaneously cloned into the pACYCDuet-1 or pETDuet-1 plasmids to achieve dual gene co-expression (pACYCDuet-ATA117-ADH or pETDuet-ATA117-ADH), thereby also reducing the number of bacterial cultures. Subsequently, the protein expression between the two tandemly expressing recombinant engineered bacteria was compared using the SDS-PAGE (Figure 5A). The results revealed that as compared to the pACYCDuet-ATA117-ADH recombinant engineered bacteria, the solubility of the protein expression from the pETDuet-ATA117-ADH recombinant engineered bacteria was superior. Furthermore, the amount of target protein in the supernatant of pETDuet-ATA117-ADH was found to be higher while the same in the cell pellet was found to be lower, which is more in line with the demand. These clearly indicate that genes located in pET plasmids had much better expression levels than those located in pACYC plasmids, a conclusion consistent with that reported by H. Chen et al. [41]. For the multi-gene cluster, the up-stream genes usually have higher expression levels than those downstream genes [42], thus arranging the *ATA117* gene before the *ADH* gene.

By pairing two recombinant plasmids with different antibiotic resistance markers, *E. coli* BL21(DE3) cells successfully transformed with the two plasmids were screened on a plate containing respective antibiotics. As plasmids with similar traits usually have the same or similar replication regulation mechanisms, their repressors (repressors or repeats) also tend to be similar. Furthermore, during regulation, they can also randomly combine with the replication region of any plasmid to inhibit the replication process, thereby resulting in its loss from the progeny cells. Accordingly, this fact is believed to be the reason why the pETDuet-ATA117 and pET28a-ADH combinations had a weaker growth rate than that of the other combinations. Our studies showed that when other combinations reached an OD_600_ of 0.8 in 3 h, pETDuet-ATA117/pET28a-ADH combination took 4 h to reach the same OD_600_. Moreover, the three dual-plasmid bacteria exhibited a certain degree of expression after being induced by their respective optimal temperatures, while the amount of the target protein was not significantly different (Figure 5B). Additionally, the ADH fusion protein (39.5 kDa) is 0.5 kDa larger than the ATA117 fusion protein; hence, there is a higher possibility of their bands getting partially overlapped during SDS-PAGE. Accordingly, considering the stability and specific activity of the plasmids (Table 2), pACYCDuet-ATA117/pETDuet-ADH was considered the best choice among the three. In order to further verify that both ATA117 and ADH can be expressed by recombinant engineering bacteria, the induced bacterial cells were sonicated and the supernatant was collected. Subsequently, the protein concentration was determined using the Bradford method. The results showed that the co-expressing bacteria could express the two proteins normally (Table 2). Additionally, when used in the asymmetric amination reaction of 207.8 mM BPO, all the five ATA117 and ADH co-expressing recombinant plasmid strains achieved a high enantiomeric excess (99%). Notably, the specific activity of ATA117 of the *E. coli* BL21(DE3)/pETDuet-ATA117-ADH recombinant plasmid-engineered strain with the best specific activity among the five was determined to be 1.63 times that of the pACYCDuet-ATA117/pETDuet-ADH engineered strain. 

#### 2.4.3. Comparison of Asymmetric Synthesis of R-BPA Catalyzed by Free Bienzyme and Co-Expression Engineered Bacteria

When substrate concentration was 166.6 mM, the yield reached 99.9% at 40 °C by the mixture of two free enzymes involving ATA117 and ADH, while 82.9% yield was obtained with single enzyme ATA117 as catalyst. Furthermore, when the substrate concentration increased to 207.8 mM, the yield decreased to 91.7% by the mixture of two free enzymes, while the yield reached 99.9% and 91.8% by the whole cell *E. coli* BL21(DE3)/pETDuet-ATA117-ADH and *E. coli* pETDuet-ATA117/pET28a-ADH, respectively. The yield reached 83.1%, 69.6%, and 56.2% with the whole cell *E. coli* pACYCDuet-ATA117/pETDuet-ADH, *E. coli* pACYCDuet-ATA117/pET28a-ADH, and *E.* coli pACYCDuet-ATA117-ADH (Table 4). Therefore, it can be inferred that the catalytic activity of *E. coli* pACYCDuet-ATA117/pETDuet-ADH was slightly higher than that of pACYCDuet-ATA117/pET28a-ADH. In general, pETDuet-ATA117-ADH was the best co-expression system among the five co-expression systems, and the yield reached 99.9%. 

## 3. Materials and Methods

### 3.1. Materials

Kanamycin (Kan) and PLP were purchased from Shanghai Yien Chemical Technology Co., Ltd., Shanghai, China. Isopropyl-β-D-thiogalactopyranoside (IPTG) and isopropylamine hydrochloride (IPA) were purchased from Shanghai Aladdin Biochemical Technology Co., Ltd., Shanghai, China. Ampicillin (Amp) was purchased from Shanghai Macklin Biochemical Co., Ltd., Shanghai, China. Restriction endonucleases were purchased from Bao Ri Doctor Biotechnology Co., Ltd., Beijing, China. NADH and BeyoGold™ His-tag Purification Resin (resistance to reduction and chelation) were purchased from Shanghai Biyuntian Biotechnology Co., Ltd., Shanghai, China. Sodium chloride, tryptone, and yeast extract were purchased from Shanghai Shenggong Biological Engineering Technology Service Co., Ltd., Shanghai, China, while 3,5-bis(trifluoromethyl)acetophenone (98%) was purchased from Jiangsu Aikang Biomedical R&D Co., Ltd., Changzhou, China. S-BPA and R-BPA standards were purchased from Shanghai Bide Pharmaceutical Technology Co., Ltd. Shanghai, China. *Escherichia coli* BL21(DE3) host bacteria were purchased from Beijing Qingke Biotechnology Co., Ltd. (Shanghai, China). All other chemicals were of analytical grade and were purchased from Sinopharm Chemical Reagent Co., Ltd. (Shanghai, China).

### 3.2. Analytical Methods

The yield and enantiomeric excess of R-BPA were analyzed using a Shimadzu GC-2014 gas chromatograph (GC). The sample was analyzed by GC equipped with an Agilent CP7502 J&W CP-Chirasil-Dex CB chiral column (Machery-Nagel; 25 m × 0.25 mm × 0.25 mm). Injector, column, and FID temperature were 250, 110, and 250 °C. H_2_ pressure: 83 kPa. Split ratio: 1:15. Retention time of BPO, S-BPA, and R-BPA were 3.165 min, 4.940 min, and 5.473 min. Equations (1) and (2) evaluated the yield (*X*) and enantiomeric excess of R-BPA (*ee_p_*).

(1) $$X(\%)=\frac{P\times Ms}{Q\times Mp}\times 100\%$$

*Ms* and *Mp* are the molecular weight of the substrate and the product. *P* and *Q* stand for the mass of the product at the end of the reaction and the initial mass of the substrate.

(2) $$ee_{p}=\frac{C_{R}-C_{S}}{C_{R}+C_{S}}\times 100\%$$

*C_R_* and *C_S_* stand for the concentration of R-BPA and S-BPA.

### 3.3. Expression and Purification of ATA117 and ADH from Single Expression System

The gene sequence of ATA117 (GenBank: JA717225) was obtained from the National Center for Biotechnology Information and was fully synthesized by Shanghai Sangon Bioengineering Co., Ltd., Shanghai, China. The target gene was constructed using the cloned plasmid pUC57. *ATA117* was amplified according to the method described in Table 1, and the IPTG-inducible pETDuet-1 expression vector and the *ATA117* amplified product were digested with BamHI (15 U/µL) and XhoI (10 U/µL) restriction enzymes for 3 h. The resulting enzyme-linked product of purified linearized pETDuet-1 and the fragment of *ATA117* was transformed into *Escherichia coli* BL21(DE3). BL21(DE3)/pETDuet-ATA117 positive clones were identified using colony PCR and gene sequencing. The obtained transformants were cultured in Luria Bertani (LB) medium containing 50 µg/mL Kan at 180 rpm and 37 °C until the OD600 reached ~0.8. The gene expression was induced according to the conditions listed in Table 1. Finally, the overexpressing cells were collected after induction by centrifugation for 10 min at 8000 rpm and 4 °C. The cells were then washed twice with 0.9% (*v*/*w*) saline [43]. The harvested cells were resuspended in 100 mM sodium phosphate buffer (PBS, pH 7.0) at 50 g/L, and the mixture was sonicated for 5 min (power 400 W, worked for 3 s and spaced for 7 s). The supernatant and precipitation of the lysate were collected by centrifugation at 8000 rpm and 4 °C for 10 min. The expression of the target enzyme was judged by 10% sodium dodecyl sulfate polyacrylamide gel electrophoresis (SDS-PAGE). The crude ATA117 enzyme solution was subsequently purified using BeyoGold His-tag purification resin (Beyotime Biotechnology., Beijing, China). The protein concentration was measured using the Bradford assay. Protein expression and molecular weight were determined by 10% SDS-PAGE. The protein bands were visualized using Coomassie blue stain. Other recombinant plasmids, such as BL21(DE3)/pET28a-ATA117 were induced and purified using similar methods. 

*ADH* was codon-optimized and synthesized with an N-terminal His-tag by Beijing Qingke Biotechnology Technology Co., Ltd., Beijing, China. The gene was then inserted into an IPTG-inducible pET28a expression vector at BamHI/XhoI restriction sites. The plasmid was transformed into *E. coli* BL21(DE3) cells, which were cultivated at 37 °C and 180 rpm in LB liquid medium (pH 7.0) supplemented with 50 µg/mL kanamycin and cultured overnight. Expression was induced according to the conditions shown in Table 2. Extraction and purification of the ADH crude enzyme solution were similar to those employed for ATA117.

### 3.4. Enzyme Activity Assays

#### 3.4.1. Activity of ATA117

The reaction system employed for the ATA117 enzyme assay was composed of 832.8 mM IPA, 1 mM PLP, 0.3 mL DMSO containing 166.6 mM BPO, 1 mL of fermentation broth centrifuged cells or broken cell enzyme solution, and 100 mM Tris-HCl (pH 9.0) in a total volume of 2 mL. The reaction was performed at 40 °C and 180 rpm for 2 h and stopped by the addition of 1 µL trifluoroacetate (1%). The BPA products were analyzed by gas chromatography. Under the above reaction conditions, one unit (U) of enzyme activity was defined as the amount of enzyme required to produce 1 µmol of R-BPA per min under standard assay conditions. 

#### 3.4.2. Activity of ADH

The enzyme activity determination system for ADH comprised of glycine-sodium oxide buffer (pH 8.6), 1 × 10^−3^ mol/L NAD^+^, and 5 × 10^−3^ mol/L ethanol in a total volume of 5 mL. After warming at 40 °C in a water bath for 20 min, the enzyme solution that was warmed under the same condition was added. After 5 min, the absorbance was measured at 340 nm every 1 min. The amount of enzyme required for increasing the A_340_ by 0.001 per min at 40 °C and pH 8.6 was considered to be one unit (U). 

### 3.5. Catalytic Properties of Purified ATA117 Expressed by the E. coli BL21(DE3)/pETDuet-ATA117 Recombinant Engineering Bacteria

#### 3.5.1. Substrate Specificity and Enantioselectivity

The substrate scope of TAs can be considered relatively broad because they are capable of catalyzing the amination of a wide range of aldehydes and ketones. However, the active sites of these enzymes contain small and large binding pockets; as a result, wild-type enzymes are restricted to ketones bearing at least one ‘small’ substituent (methyl or ethyl). Considerable effort has been directed toward increasing the capacity of the small binding pocket for enabling these enzymes to accept ketones with two bulky substituents; ATA117 is one such successful example. Accordingly, to examine the substrate specificity of ATA117, the reactivity of 13 amino acceptors was compared (Table 3). 

#### 3.5.2. Effects of pH and Temperature on Enzyme Activity

The optimum pH for the reaction with 832.8 mM IPA and 166.6 mM BPO was determined at 40 °C using different buffer systems (100 mM) in the pH range of 6.0 and 10.0. Accordingly, sodium phosphate buffer (pH 6.0–8.0), Tris-HCl (pH 8.0–9.0), and glycine-NaOH (pH 9.0–10.0) were used for this purpose. To investigate the effect of temperature on the enzyme activity, the reaction was carried out at different temperatures ranging from 30–50 °C at pH 9.0, using IPA as the amino donor and BPO as the amino acceptor.

#### 3.5.3. Effects of Metal Ions and Reagents on Enzyme Activity

The effects of metal ions and reagents on ATA117 activity were studied by pre-incubating the enzyme with the metal ions and reagents in Tris-HCl buffer (0.1 M, pH 9.0) for 1 h at 40 °C. The final concentration of Ca^2+^, Mg^2+^, Cu^2+^, Fe^2+^, Zn^2+^, Mn^2+^, Na^+^, K^+^, and EDTA was 10 mM, and the activity of ATA117 was determined under standard conditions. The relative activity (%) was calculated using the activity determined without adding any metal ions as a control (100%).

#### 3.5.4. Comparison of ATA117 Activity from *E. coli* BL21(DE3)/pETDuet-ATA117 Whole Cells and Crude Enzyme Solution

After 15 h of induction at 18 °C, the overexpressed cells were collected by centrifugation at 8000 rpm and 4 °C for 10 min. The cell pellets were washed twice with 0.9% (*v*/*w*) saline and resuspended in 100 mM sodium phosphate buffer (pH 7.0). The suspension was divided into two equal parts. Subsequently, half of the volume of the resuspension was removed and subjected to ultrasonic disruption (power 400 W, worked for 3 s, and spaced for 7 s) for 5 min at 4 °C. The cell suspension was then centrifuged at 8000 rpm for 10 min. After centrifugation, the supernatant was used as a crude enzyme solution. The enzyme activity determination method described in Section 2.4.1 was used to determine and compare the activity of the crude enzyme solution and the whole-cell catalytic reaction.

### 3.6. Preparation of Bienzyme Cascade System by Two Different Ways: Free Single Enzyme and Co-Expression Engineering Bacteria

The coupling system of ATA117 and ADH was divided into two main steps; (1) the amination reaction of BPO followed by (2) the in situ removal of the by-products. The latter reaction converts the acetone by-product of the former reaction into isopropyl alcohol, which improves the efficiency of the amination reaction of BPO, the substrate (Figure 1). The preparation of the bienzyme cascade system included the preparation of a bienzyme coupling system composed of ATA117 and an ADH-free enzyme along with the preparation of five double-gene co-expression systems. 

#### 3.6.1. Preparation of R-BPA by Bienzyme Cascade Reaction with Free Enzyme Mixture Containing ATA117 and ADH

Substrate BPO (166.6 mM substrate BPO dissolved in 0.3 mL cosolvent DMSO), 832.8 mM amino donor IPA, 1 mM cofactor PLP, 1 × 10^−3^ mol/L cofactor NADH, 18 mg ADH (0.15 U/mg), and 15 mg ATA117 (0.18 U/mg) were added to the reaction system, and 0.1 M Tris-HCl buffer (pH 9.0) was used to supplement the system to form a cascade reaction group. The reaction system was maintained at 30, 35, 40, 45, and 50 °C for 24 h at 180 rpm. A system without ADH was used as the blank control group. Considering the effect of substrate solubility, various cosolvents, such as methanol, ethanol, Tween 80, Tween 20, Span 80, glycerol, and DMSO, were additionally evaluated for their effects on the cascade reaction of the bienzyme system.

#### 3.6.2. Preparation of R-BPA by Bienzyme Cascade System via Co-Expression Engineering Bacteria Constructed by Two Double-Gene Tandem Co-Expression Systems

Engineered BL21(DE3)/pETDuet-ATA117-ADH and BL21(DE3)/pACYCDuet-ATA117-ADH were expressed under their respective optimal induction conditions (Table 2). After induction, the fermentation broth was centrifuged at 8000 rpm and 4 °C for 10 min to remove the medium components. The cells were then washed three times with an appropriate amount of normal saline to ensure that the medium components were removed. The treated wet cells (0.25 g) were resuspended in 0.1 M Tris-HCl buffer (pH 9.0), and the system was made up to 1.4 mL. Substrate BPO (207.8 mM substrate BPO dissolved in 0.3 mL cosolvent DMSO), 1038.8 mM amino donor IPA, and 1 mM cofactor PLP were added to the above system, and 2 mL of the reaction system was made up of 0.1 M Tris-HCl buffer (pH 9.0). The reaction system was maintained at 40 °C and 180 rpm for 24 h. 

#### 3.6.3. Preparation of R-BPA by Bienzyme Cascade System via Co-Expression Engineering Bacteria Constructed by Two-Plasmid Co-Expression System

The BL21(DE3)/pACYCDuet-ATA117 and BL21(DE3)/pET28a-ADH strains were cultured in a 37 °C/180 rpm constant-temperature incubator until the OD_600_ reached 0.8. Next, 10 µL of the purified pACYCDuet-ATA117 and pET28a-ADH plasmids were transformed into BL21(DE3) competent cells, simultaneously. After activation for 1 h, the cells were spread on LB solid medium containing two antibiotics (Cm_34_ and Kan_50_), and the culture was inverted overnight to screen the positive clones containing the two recombinant plasmids. Using the same method, the purified pETDuet-ATA117 and pET28a-ADH plasmids were transformed into BL21(DE3) cells similar to the pACYCDuet-ATA117 and pETDuet-ADH plasmids. It should be noted that the medium must be screened by the simultaneous addition of antibiotics to both plasmids. The construction and culture conditions of *E. coli* transformed with pETDuet-ATA117 and pET28a-ADH have been mentioned in Section 3.3 and hence, not mentioned here again. The construction and culture conditions of the other two recombinant plasmids, pACYCDuet-ATA117 and pETDuet-ADH, required for the construction of double-plasmid engineered bacteria are listed in Table 2. After induction, the fermentation broth was centrifuged at 8000 rpm and 4 °C for 10 min to remove the medium components. The cells were then washed three times with an appropriate amount of normal saline to ensure that the medium components were removed. The treated cells (0.25 g) were resuspended in 0.1 M Tris-HCl buffer (pH 9.0) and the system was made up to 1.4 mL. BPO substrate (207.8 mM) dissolved in 0.3 mL cosolvent DMSO, 1038.8 mM amino donor IPA, and 1 mM cofactor PLP were added to the above system. Finally, 2 mL of the reaction system was made up with 0.1 M Tris-HCl buffer (pH 9.0). The reaction system was maintained at 40 °C and 180 rpm for 24 h.

## 4. Conclusions

Biocatalysis has a proven track record in providing alternatives to individual chemical reactions with less impact on the environment. Cascade reactions are an extension of biocatalysis that couples a series of reactions and thus can provide alternatives to multiple chemical steps. Cascade reactions also offer several other advantages over conventional biocatalysis. In particular, they provide a way to utilize enzymes that are close to equilibrium or have unstable substrates or products. In a cascade reaction, side reactions are also minimized as successive reactions that push the equilibrium in favor of the desired product.

In the present study, 50 g/L R-BPA was synthesized using *E. coli* BL21(DE3)/pETDuet-ATA117-ADH as the best catalyst because of its excellent yield (99.9%) and high enantiomeric excess (99.9%). To the best of our knowledge, this is the first report utilizing the combined application of ATA117 and ADH in the production of R-BPA to achieve more efficient biological transamination. This method is suitable for the amine transfer reaction with IPA as the donor. On this basis, it can be applied to amination of a variety of substrate ketones, such as acetophenone series compounds. In this study, we have demonstrated the unique advantages and complementary potential of the bienzyme pair in an established cascade system, with significantly improved substrate handling capacity and specific activity of bienzyme cascade system. The current study thus provides a feasible and comprehensive strategy to combine ATA117 and ADH for the efficient production of chiral amines with engineered bacteria in addition to providing valuable insights into the synergy of bienzymes in biocatalysis.

## Figures and Tables

**Table 1:** PCR process for the construction of recombinant plasmids.

	Vectors	Target Gene	PCR Template	Primer	Sequence 5′ → 3′	Restriction
single gene part	pETDuet-1	ATA117	PUC57-ATA117	pETDuet-ATA-F	CGGGATCCGATGGCGTTCTCAGCGGACACCC	BamHI
				pETDuet-ATA-R	TTCCGCTCGAGGTACTGTACCGGGGTCAGCC	XhoI
	pET28a	ATA117	pETDuet-ATA117	pET28a-ATA117-F	CGGATCCATGGCGTTCTCAGCGG	BamHI
				pET28a-ATA117-R	GCTCGAGGTACTGTACCGGGGTCAG	XhoI
	pETDuet-1	ADH	pET28a-ADH	pETDuet-ADH-F	AGCCAGGATCCGATGAGCATTCCGG	BamHI
				pETDuet-ADH-R	CAGACTCGAGTTTGCTGGTATCAACAAC	XhoI
co-expression part	pETDuet-1	ATA117	pETDuet-ATA117	pETDuet-ATA117-F_4_	cacagccaggatccgaattcgATGGCGTTCTCAGCGGACA	EcoRI
				pETDuet-ATA117-R_3_	gcggccgcaagcttgtcgacGTACTGTACCGGGGTCAG	SalI
	pETDuet-ATA117	ADH	pET28a-ADH	pETDuet-ATA117-ADH -F_3_	agaaggagatatacatatgATGAGCATTCCGGAAACCCAGA	NdeI
				pETDuet-ATA117-ADH-R_3_	gtttctttaccagactcgagTTTGCTGGTATCAACAACATAACGA	XhoI
	pACYCDuet	ATA117	pETDuet-ATA117	pACYCDuet-ATA117-F_2_	cacagccaggatccgaattcGATGGCGTTCTCAGCGGACA	EcoRI
				pACYCDuet-ATA117-R_2_	gcggccgcaagcttgtcgacGTACTGTACC GGGGTCAG	SalI
	pACYCDuet-ATA117	ADH	pET28a-ADH	pACYCDuet-ATA117-ADH -F_2_	agaaggagatatacatatgATGAGCATTCCGGAAACCCAGA	NdeI
				pACYCDuet-ATA117-ADH-R_2_	gtttctttaccagactcgagTTTGCTGGTATCAACAACATAACGA	XhoI

**Table 2:** Induced expression of the constructed recombinant plasmids.

Enzyme Expression Form		Original Plasmid	Recombinant Plasmid	Growth Temperature (°C)	Optimum Induction Temperature (°C)	Optimum Inducer Concentration (mmol/L)	Optimal Induction time (h)	Protein Concentration (mg/mL)	Specific Activity of ATA117 (U/mg)	Specific Activity of ADH (U/mg)
single enzyme	ATA117	pETDuet	pETDuet-ATA117	37	18	1	15	0.19	0.18	/
		pACYCDuet	pACYCDuet-ATA117	37	18	1	16	0.11	0.09	/
		pET28a	pET28a-ATA117	37	18	1	12	0.15	0.14	/
	ADH	pETDuet	pETDuet-ADH	37	18	0.5	15	0.20	/	0.16
		pET28a	pET28a-ADH	37	30	1	12	0.16	/	0.15
bienzyme	ATA117/ADH co-expression	pETDuet	pETDuet-ATA117-ADH	37	18	1	8	0.37	0.18	0.15
		pACYCDuet	pACYCDuet-ATA117-ADH	37	18	1	8	0.21	0.09	0.08
		pETDuet and pET28a	pETDuet-ATA117/pET28a-ADH	37	23	1	14	0.34	0.12	0.10
		pACYCDuet and pET28a	pACYCDuet-ATA117/pET28a-ADH	37	23	1	14	0.26	0.11	0.15
		pACYCDuet and pETDuet	pACYCDuet-ATA117/pETDuet-ADH	37	18	1	15	0.31	0.11	0.16

**Table 3:** Screening of substrate spectrum.

Substrate	Yield (%)	e.e. (%)
Acetophenone	44.49	72.7
Benzyl acetone	73.56	22.4
P-methoxypropiophenone	55.38	32.45
3,5-bistrifluoromethylacetophenone	100	100
Ethyl levulinate	0	0
Ethyl 4-acetobutyrate	2.35	100
Methyl acetoacetate	100	91
Ethyl acetoacetate	100	73.59
Tert-Butyl acetoacetate	61	77
Sitagliptin precursor ketone	100	100
Ethyl benzoyl formate	0	0
Ethyl benzoylacetate	0	0
Ethyl 2-oxo-4-phenylbutyrate	0	0

Reaction system: substrate 43 mM, isopropylamine hydrochloride 215 mM, PLP 1 mM, 15% DMSO, 1 mL of 15 mg/ mL transaminase pure enzyme aqueous solution (total enzyme activity 2.7 U), 0.1 M pH9 Tris-HCl to make up 2 mL volume.

**Table 4:** Comparison of asymmetric synthesis of R-BPA catalyzed by free bienzyme and co-expression engineered bacteria.

Catalyst		Substrate Concentration (mM)	Yield (%)	Enantiomeric Excess (%)
Free enzyme	the mixture of ATA117 and ADH	166.6	99.9	99.9
		207.8	91.7	99.9
	ATA117 single enzyme	166.6	82.9	99.9
		207.8	73.8	99.9
Whole cell	*E. coli* BL21(DE3)/pETDuet-ATA117-ADH	207.8	99.9	99.9
	*E. coli* BL21(DE3)/pACYCDuet-ATA117-ADH	207.8	56.2	99.9
	*E.coli* BL21(DE3)/pETDuet-ATA117/pET28a-ADH	207.8	91.8	99.9
	*E. coli* BL21(DE3)/pACYCDuet-ATA117/pET28a-ADH	207.8	69.6	99.9
	*E. coli* BL21(DE3)/pACYCDuet-ATA117/pETDuet-ADH	207.8	83.1	99.9

## Data Availability

All data generated or analyzed during this study are included in this article.
